# Short-Term Enrichment Makes Male Rats More Attractive, More Defensive and Alters Hypothalamic Neurons

**DOI:** 10.1371/journal.pone.0036092

**Published:** 2012-05-02

**Authors:** Rupshi Mitra, Robert M. Sapolsky

**Affiliations:** 1 School of Biological Sciences, Nanyang Technological University, Singapore, Singapore; 2 Division of Psychology, Nanyang Technological University, Singapore, Singapore; 3 Department of Biology, Stanford University, Stanford, California, United States of America; University of Salamanca- Institute for Neuroscience of Castille and Leon and Medical School, Spain

## Abstract

Innate behaviors are shaped by contingencies built during evolutionary history. On the other hand, environmental stimuli play a significant role in shaping behavior. In particular, a short period of environmental enrichment can enhance cognitive behavior, modify effects of stress on learned behaviors and induce brain plasticity. It is unclear if modulation by environment can extend to innate behaviors which are preserved by intense selection pressure. In the present report we investigate this issue by studying effects of relatively short (14-days) environmental enrichment on two prominent innate behaviors in rats, avoidance of predator odors and ability of males to attract mates. We show that enrichment has strong effects on both the innate behaviors: a) enriched males were more avoidant of a predator odor than non-enriched controls, and had a greater rise in corticosterone levels in response to the odor; and b) had higher testosterone levels and were more attractive to females. Additionally, we demonstrate decrease in dendritic length of neurons of ventrolateral nucleus of hypothalamus, important for reproductive mate-choice and increase in the same in dorsomedial nucleus, important for defensive behavior. Thus, behavioral and hormonal observations provide evidence that a short period of environmental manipulation can alter innate behaviors, providing a good example of gene-environment interaction.

## Introduction

Environmental enrichment (EE) is known to improve physiology and behavior at multiple levels in a variety of species. In rodents, early post-natal EE reduces emotional reactivity [1] and enhances hippocampal-dependent learning in adults [2]. EE protects against age dependent cognitive decline [3], [4]. It additionally reduces lesion size and behavioral deficits post-stroke in young and aged rats [5], [6]. EE also lessens many adverse effects of stress, instead promoting homeostatic coping [4], [7], [8] and is known to be anxiolytic [9]. Interestingly, EE successfully restores behavioral deficits produced through purely genetic means [10], [11]. EE in early development increases overall thickness in a variety of cortical regions, as well as of different individual cortical layers [2], [12], [13], [14], [15]. At the level of cellular structure and function, EE stimulates neurogenesis in the adult brain [16], [17], is neuroprotective [18], and enhances synaptic plasticity [19], [20], [21], [22]. Furthermore, EE increases expression of neurotrophins such as NGF and BDNF [23], [24], [25], [26]. These protective molecular effects also extend to genes important for learning and memory [10]. Finally, EE in humans can reduce lesion size post-stroke [27], [28], [29], [30].

This large body of evidence suggests that EE generally promotes the well being of an organism and is protective against insults. Most of these studies have focused on laboratory paradigms of learning and fear, and of their perturbations. However, the effects of EE on ethologically relevant naturalistic behaviors remain largely unexplored. In this report we investigate the effects of EE on two salient naturalistic behaviors: intra-species signaling in form of mate choice and inter-species signaling in form of aversion to predator odors. Both of these responses are strong and hard-wired, reflecting intense selection pressure for their maintenance. Biological mediators of mate choice are often inter-twined with predation risk. For example, bright plumage of males of many bird species enhances reproductive attractiveness in parallel with making animals more visible to predators. It can be plausibly argued that a male rat signaling its resource-rich physiological status by pheromones will also invite unwanted attention from predators “eavesdropping” on chemical signals. Hence, we tested if enriched males engage in compensatory behavioral modifications to reduce their predation risk. We show that short-term sensory enrichment makes males more attractive to a receptive estrous female and induces stronger aversion to predator odor in them.

Castration of males abolishes their attractiveness to females, which is reinstated if castrated males are supplemented with exogenous testosterone [31]. This suggests that production of pheromones and hence attractiveness to females in rats is dependent, at least in part, on testosterone. On the other hand, the stress hormone corticosterone is secreted during defensive response [32]. Thus we measured both testosterone and corticosterone levels. Both reproductive attraction and innate fear are driven by specific nuclei of the ventromedial hypothalamic zone (VMH). Specifically, the dorsomedial nucleus (VMHdm) is important for defensive behaviors, while the ventrolateral nucleus (VMHvl) is important for reproductive behaviors [33]. Structural plasticity of dendrites has important implications for functional plasticity through its effects on electrotonic properties, incorporation of active channels and formation of new synapses. Moreover, dendritic changes are often associated with behavioral changes [34], [35], [36]. Thus, we tested if neurons of VMHdm and VMHvl undergo correlated changes that might drive any observed behavioral changes.

## Materials and Methods

### Ethics Statement

All procedures related to animal maintenance and experimentation were approved by the Stanford University's Administrative Panel on Laboratory Animal Care (APLAC) and were in accordance with animal care standards outlined in National Institute of Health (USA) guidelines.

### Animals and Experimental groups

Adult male Wistar rats (10 weeks, Charles River, Wilmington, MA) were housed in standard laboratory cages (3 animals per cage) with food and water *ad libitum* and a day-night cycle of 14:10 h (lights on at 7 am). After 2–3 days of habituation in standard cages, animals were divided into enriched (EE) and non-enriched groups. EE composed of 14 days of housing in a highly sensory environment as described before [8]. In short, animals (3/cage) were housed in a bigger cage (dimension; 60 cm×60 cm×60 cm) compared to standard laboratory cage (45 cm×24 cm×20 cm) with wire-net walls, cylindrical burrowing tubes, climbing plank, objects, nesting material and fruit-flavored chews. The objects were rearranged and orientation of burrows and planks were altered every 4^th^ day. Non-enriched animals continued to be housed in standard laboratory rodent cages (3/cage). During the course of experiments, control and enriched animals gained comparable body weight (control: 58.9±4.4 g and enriched: 59.6±3.5 g; *p*>0.9, student's t-test). Separate cohort of animals was used for different behavioral tests.

### Mate choice

Estrous stage of female was determined based on cytological features (cornified cells) of vaginal lavage. For Mate-choice, one adult female animal in estrus was introduced into the centre of a rectangular arena (92×35 cm, 40 cm high). Two standard mouse cages with enriched and non-enriched males were placed at terminal ends of the arena. The cages consisted of original bedding from each of the groups. Females were changed between each session for different pair of male animals. The total time of exploration of the arena by the female was 10 minutes. The amount of time the female spends near each of the male cages were monitored and calculated as percentage time spent in the male arena. This represents the female's preference for each of the males in the mate-choice task. A total of 25 females were used for mate-choice test and the test was done in morning (8am–12noon) and afternoon (1–6pm). Video records were analyzed using Any-Maze software (Stoelting, Wood Dale, IL, USA). Some of video records were analyzed manually using stop-watch in view of lower contrast. Video records were coded before the analysis and experimenter was blind to treatment conditions being analysed.

### Aversion to predator odor

For predator odor test, both group of males (6 per group) were exposed individually to a rectangular arena consisting of predator odor placed in a towel at one end of arena (92×35 cm, 40 cm high; 2 ml bobcat urine placed on a black piece of 6 cm×6 cm towel). They were allowed to explore the arena for 10 minutes and time spent in bisect containing predator odor was scored. Percentage time spent represented preference for predator odor. Additionally, time spent exploring the towel containing predator odor was also monitored as preference for predator odor. Risk assessment was measured as the number of stretch-attend posture the animal showed while exploring the area near predator odor. This task specifically represents an active effort taken by the animal to assess the amount of risk present in a particular area [37], [38]. The test was carried out in morning (8am–12noon).

### Behavior in elevated plus maze and open arena

Both groups (6 animals per group) were allowed to explore novel objects (ceramic objects) in a square arena (60 cm×60 cm×45 cm) for 5 minutes and total time of exploration (in seconds) was monitored. Anxiety-like behavior was tested in an elevated plus maze (9 animals per group; 5 minute each trial), consisting of two threatening (open and illuminated; each arm 60 cm×15 cm) and two non-threatening arms (walled and dark). Relative exploration in “open” arms, i.e., percentage time spent in open arm and each sortie (total time spent in open arm/number of entries) in open arm was quantified as index of anxiety [36]. Two different cohorts of animals were used for the two different tests. The tests were carried out in morning (8am–12noon).

### Endocrine measurement

For testicular measurements, one testis of each animal was homogenized and suspended in diethyl ether (twice the volume of homogenized testis sample). The mixture was shaken and layers were allowed to separate. Top layer containing the solvent was pipetted out in a clean test tube. The procedure was repeated three times and solvent layers were combined. Diethyl ether was then evaporated to dryness. Extracted testosterone was dissolved in one ml of aqueous buffer. Testosterone concentration was estimated by enzyme linked immunoassay using commercial kit (Assay Designs, Ann Arbor, MA, USA).

Testosterone and corticosterone concentration in plasma was also measured. Both testosterone and corticosterone were measured in males at three different stages, at basal level (12 animals per group), following exposure to estrous female (6 animals per group) and following exposure to predator odor (6 animals per group). Measures for basal and post female exposure were taken from the same animal on the same day with a gap of at least 3 hours. Measure for predator odor was taken on the next day. Tail vein blood was collected 30 minutes post-exposure and prepared for endocrine measurement as described before [36]. In short, blood was heparinised and centrifuged (centrifuge model 5415C, Eppendorf) at 10,000 rpm for 10 min to obtain plasma. Titers (CORT and Testosterone) were assessed by using a competitive enzyme immune assay kit (Assay Design). Typical values for sensitivity and coefficient of variation for testosterone EIA kit are 5.67 pg/ml (range 7.81–2,000 pg/ml) and 7.8% to 10.8%, respectively. Typical values for sensitivity and coefficient of variation for corticosterone EIA kit are 27.0 pg/ml (range 32–20,000 pg/ml) and 6.6% to 8%. Animals used for blood collection were not used for behavioral tests to avoid any influence of blood collection on behavior.

### Morphological analysis

Separate group of animals (4–6 per group) were used for morphological analysis to rule out any influence of behavior or endocrine measurement on neuronal plasticity. Following 14 days of EE both group of animals were sacrificed under deep anesthesia and brain tissue was collected and processed with rapid Golgi staining technique described earlier [36]. In short, brain tissue containing the VMH and medial amygdala was immersion fixed and stained using a series of solutions including potassium dichromate, formaldehyde and silver nitrate. Completely impregnated, uncut neurons were selected from VMH nuclei (ventrolateral and dorsomedial nuclei) and medial amygdala. Camera Lucida neuronal tracings (500×) were obtained (Nikon Phase Contrast) from selected neurons and were then scanned (8-bit grayscale TIFF images with 1200 dpi resolution; Canon MultipassMP360) along with a calibrated scale for subsequent computerized image-analysis. Custom-designed macros embedded in ‘NIH Image’ software were used for morphometric analysis of digitized images. Using the center of the soma as the reference point, dendritic length was measured as a function of radial distance from the soma, similar to previous report [36]. 30–40 neurons per group per area were analyzed to compare between enriched and non-enriched animals.

### Statistics

Results are reported as Mean ± SEM. Wilcoxon signed rank test was used to estimate mate choice of estrous females for two parts of arena with control and enriched male odors. Mann-Whitney test was performed to compare inter-group differences in all other instances. In both cases, exact tests were used to attain freedom from distributional assumptions. Wherever appropriate, a chi-square test was used to test deviation from ratios predicted by random occurrence.

## Results

**Figure 1:**
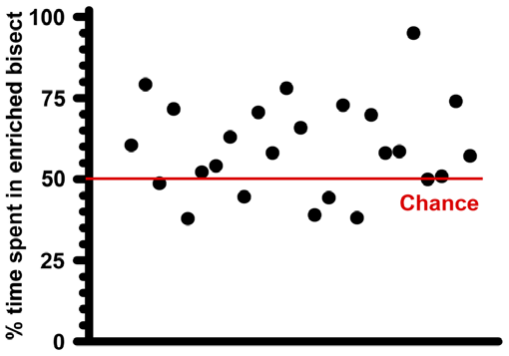
Females prefer enriched males. In a 2-choice preference task, estrous females spent more time in bisect containing inaccessible enriched males and its soiled bedding. Ordinate depicts % time spent in enriched bisect. Each dot in abscissa represents raw data from one female. (relative to total time in control plus enriched bisects). Chi^2^ test: *p*<0.015, n = 25.

**Figure 2:**
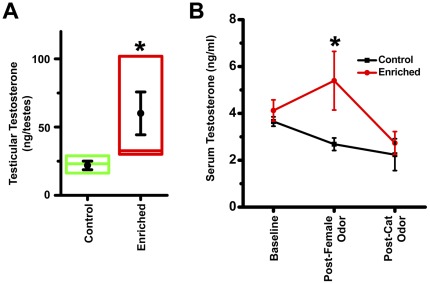
Enriched males have higher testosterone. Gonads of enriched animals contained more testosterone (A). n = 8 males for each group. Box plots all through the figure depict median, 25^th^ percentile and 75^th^ percentile. Dot and whiskers represent mean and SEM. *, *p*<0.05; exact Mann-Whitney test. Enriched animals secreted more testosterone in blood when presented with an estrous female, an effect absent in control males (B). n = 6 males for each group. *, *p*<0.05, exact Mann-Whitney test between control and enriched.

We tested the preference of sexually-naïve females for enriched males against controls. Preference was determined by comparing time spent by an estrous female in two opposing arms of an open rectangular arena; each arm containing an inaccessible male (control or enriched and its soiled bedding). Sexually naïve females spent more time in the enriched bisect (Figure 1; Wilcoxon signed rank test: *p*<0.01; 453±29 s in enriched bisect versus 306±26 s in control, n = 25 females). A preference score of enriched males was computed for each trial by dividing time spent in the enriched bisect by that in control. During 75% of trials, females spent more time in the enriched bisect (Chi^2^ test: *p*<0.015; median preference score = 1.4). We tested if EE increased testosterone levels of males in parallel to making these males more attractive. The level of testosterone inside testis was measured by enzyme-linked immune-assay. Enriched animals contained greater amount of testosterone (Figure 2a; 174% relative to control; *p*<0.05, exact Mann-Whitney test, n = 8 males in each group). The amount of circulating testosterone in the bloodstream was also quantified. Control animals did not exhibit a rise in circulating testosterone concentrations when exposed to an estrous female. Circulating testosterone concentrations were elevated in enriched animals when exposed to an inaccessible estrous female (Figure 2b, *p*<0.05, exact Mann-Whitney test, n = 6 males in each group) compared to control group. The specificity of the testosterone rise is borne out by the fact that cat odor did not raise testosterone concentrations.

**Figure 3:**
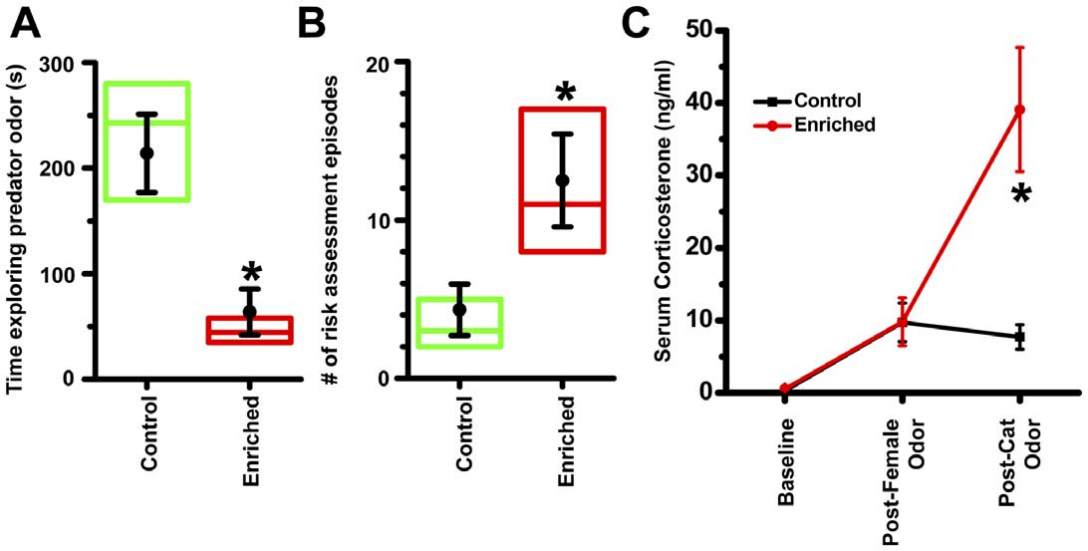
Enriched males are more defensive to predator. When presented with a towel containing bobcat urine, enriched animals spent less time in vicinity of odor (A) and engaged in greater risk assessment (B). n = 6 males for each group. Enriched animals secreted greater amount of stress hormone, corticosterone, when exposed to cat odor (C). n = 6 males for each group. *, *p*<0.05; exact Mann-Whitney test between control and enriched.

To test for predation risk posed by enrichment, control and enriched males were presented with 2 ml of bobcat urine in an arena and their explorations were recorded. Enriched animals exhibited a robust increase in defensive behaviors compared to respective controls. Enriched animals spent less time exploring the predator odor (Figure 3a; 70% reduction; *p*<0.01, exact Mann-Whitney test, n = 6 males in each group). Additionally, when exploring predator odor, enriched animals undertook more risk assessment, as demonstrated by an increased incidence of stretch attend posture (Figure 3b; 189% increase over control; *p*<0.05, exact Mann-Whitney test, n = 6 males in each group). Enriched animals exhibited more robust increase in level of the stress hormone corticosterone, as compared to control animals (Figure 3c, exact Mann-Whitney test, n = 6 males in each group), after exposure to cat. The increased corticosterone concentration in enriched animals was specific to predator threat, not occurring after exposure to an estrous female.

**Figure 4:**
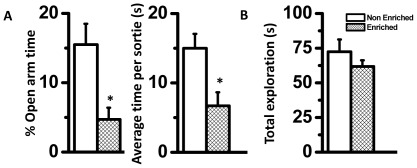
Enriched animals avoid threatening environment. Enriched animals exhibit specific avoidance of threatening open arms of an elevated plus maze, n = 7 (A). Enriched animals explore novel objects similarly as control animals (B), n = 6, *, *p*<0.05; exact Mann-Whitney test.

Lesser exploration by enriched males near predator odor was not due to a general lack of exploration. When presented with a novel stimulus, exploration difference between both groups was not statistically significant (Figure 4 b, *p*>0.05, exact Mann-Whitney test, n = 6 males in each group; exploration near two unfamiliar ceramic objects). Innate aversion and risk assessment for predator odor is an important part of defensive behavior in rats. Another equally important facet of defensive behavior is unconditioned avoidance of open and exposed areas that afford greater visibility to predators. Parallel to greater innate fear to predator cues, enriched animals also exhibited heightened aversion to open spaces. In an elevated plus-maze, enriched animals spent less time in open arms (Figure 4 a left panel, 70% reduction; *p*<0.05 exact Mann-Whitney test, n = 7 males in each group), with each sortie in open arms lasting for a shorter time than control animals (Figure 4 a right panel, 55% reduction; *p*<0.05). The close arm entries did not differ between enriched and control animals indicating similar levels of general locomotion (Number of entries in close arm; Controls: 4.66±0.5 and Enriched: 3±0.577; *p*>0.05).

**Figure 5:**
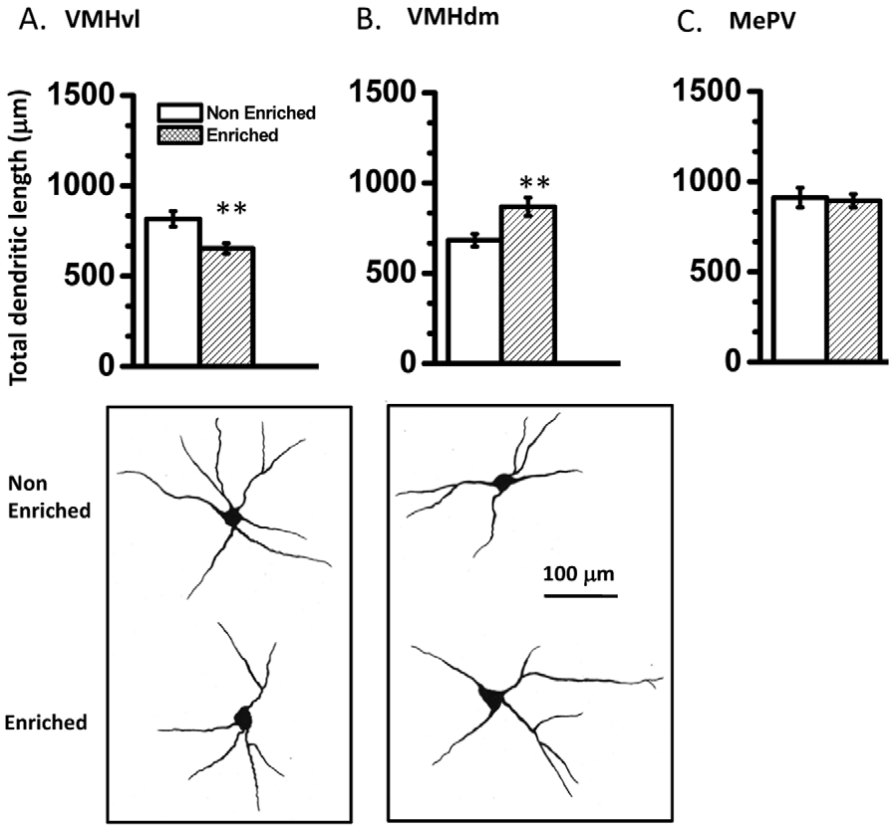
Enriched males show neuronal plasticity in hypothalamus. Enriched animals show significantly reduced dendritic length of neurons from VMHvl compared to non-enriched animals (A). Enriched animals show significantly enhanced dendritic length of neurons from VMHdm compared to non-enriched animals (B). Representative neuronal tracings from VMHvl (left) and VMHdm (right) for non-enriched (upper panel) and enriched (lower panel) animals. Scale bar is 100 µm. (*Inset*). Enriched and non-enriched animals show similar dendritic length of neurons from postero-ventral part of medial amygdala MePV (C). **p<0.01, exact Mann-Whitney test; n = 30–40 neurons per group.

In agreement with the role of VMH in innate behaviors, we observed dendritic plasticity in both sub-nuclei. Specifically, VMHvl neurons from enriched animals exhibited lower dendritic length (Figure 5A, 20% reduction; *p*<0.05 exact Mann-Whitney test, n = 33 neurons in control and 35 neurons in enriched group), while VMHdm neurons from same animals exhibited greater dendritic length (Figure 5B, 27% increase, *p*<0.05 exact Mann-Whitney test, n = 35 neurons in each group), compared to respective controls. . These differences were not carry-over manifestations of behavioral testing, because different set of animals were used for behavioral and anatomical experiments. Neurons in postero-ventral part of medial amygdala, a brain region that projects to both sub-nuclei of VMH, did not differ between control and enriched animals.

## Discussion

In this study, we show that sensory enrichment (EE) makes male rats more attractive to females. EE also leads to greater levels of gonadal androgens, which can then lead to more robust pheromone production by males [39], [40]. Additionally, enriched males enhance their defensive behaviors in what could be a behavioral compensation to greater predation risk that usually comes with more robust sexual signaling.

Female rats typically show clear mate preference [40], [41], [42]. Preference for certain males likely reflects her interest in ensuring a good genetic legacy for offspring and the avoidance of a sexually transmitted disease from a mating partner [43], [44], [45]. A male capable of securing disparate amount of resources likely has heritable capacity to endure competition from other males. It is in the interest of males to advertise their capacity through inter-sexual signaling, and it is in the interest of females to choose mates based on the intensity of these sexual signals. We report that male can advertise their access to physiological resources (e.g. testosterone) and females prefer enriched males. Because genetic legacy and access to resources are intertwined in the natural world, it is often difficult to discern causal relationships. By keeping genetic background similar through use of inbred rats, we show that EE directly influences physiological resource and mate choice.

Females choose males based on phenotypic traits that serve as markers of a male's reproductive worth [45]. Such phenotypic traits are often testosterone-dependant. In that context, it is interesting that we show enriched males produce more testosterone in their gonads and have higher circulating testosterone levels in response to exposure to a female. Change in circulating testosterone alone could imply change in either synthesis or degradation. Since here we show that testes retains the ability of make more testosterone, it suggests that change in synthesis, rather than degradation, contribute to enhanced testosterone. In agreement with our observations, female rats tend to prefer areas vacated by high-testosterone males [31].

In rodents, the intra-species signaling is mainly olfactory in nature [46], [47], [48], [49]. Being an open system, olfactory signals are very easy to be detected by eavesdropping. This results in greater opportunity costs of predation for enriched males. The predation risk can be substantial because males need to constantly re-visit their scent marks and replenish them [50]. Thus both susceptibility to predators and ability to attract mates are intimately inter-twined; increase in later necessarily increases susceptibility to predators. In this report, we show that enriched males engage in compensatory behavioral change by enhancing their innate fear to predator cues. Under laboratory conditions, aversion to predator cues manifests as lower amounts of exploration near predator odors along with frequent risk assessment episodes [37], [51]. Related to that, rats also avoid spaces that invite easy visualization by predator, showing unconditioned anxiety-like behaviors in open and illuminated places [52], [53]. Both innate fear and unconditioned anxiety-like behaviors are integral parts of the repertoire of defensive behaviors in rats, with shared ethological features, and consequently shared neural substrates [54]. In agreement with this view, our results show that EE enhances defensive behaviors in both facets of the fear repertoire. Despite being aversive, fear is an adaptive drive, in that it provides motivation and energy to a rat to actively avoid predators [54]. Rats that do not show appropriately intense fear of predators are more likely to die; thus there is immense selection pressure to maintain fear response. Hence, increase in fear by EE should be viewed as an adaptive response. It is noteworthy that adaptive value of anxiety, like any other trait, depends on prevailing environment. For example, stressful environment promotes lower maternal grooming in rats, which results in greater anxiety in progeny [55], [56]. It can be plausibly argued that these effects are adaptive for progeny as they increase vigilance in an environment expected to be filled with danger [55], [56]. In case of enriched animals, it can be speculated that their greater sexual conspicuousness increases their risk of predation, in parallel to attracting more females. Thus, high anxiety can be speculated to be adaptive in this situation.

There are substantial differences between defensive behaviors exhibited by wild rodents and domesticated laboratory strains [57]. In view of these differences, it is possible that laboratory housing environment of selective breeding diminishes fear and that EE merely restores defensive behaviors to pre-domestication baseline. These possibilities cannot be resolved using current data and need direct comparison of EE effects in wild rat populations in future. In laboratory animals, enrichment is generally thought to reduce anxiety and fear [58], [59], [60], [61], [62], contrary to the present report. These differences are probably due to the fact that we used a relatively shorter paradigm (14 days as opposed to 3–6 months) and also because we used a sensorial enrichment paradigm rather than social enrichment. However, we do not observe greater secretion of corticosterone in enriched animals at basal level. Hence, it is unlikely that our enrichment paradigm enhances fear and anxiety through induction of stress.

Pheromonal signaling in rodents can be perceived by both their own species and others as well. There are numerous examples whereby pheromonal signals are exploited by “illegitimate receivers”, including prey, predators and parasites [63]. Thus, the benefit of producing odors must be balanced with the cost of predation. Hypothetically, this should lead to simultaneous regulation of pheromone production and anti-predator response; we observe evidence for this hypothesis.

It is remarkable that relatively short periods of EE can modulate both of these behaviors. EE is known to have rather strong effects on behaviors [1], [2], [5], [7], [8], [10], [11], [20], [25]. For example, a very early study by Cooper and Zubek found that EE can annul behavioral differences established through several generation of selective breeding [64]. Laboratory strains of rats have undergone protracted selective breeding in order to make them docile. As a side-effect of selective breeding, most laboratory strains show a blunted defensive response [57], [65]. This is borne out by our observations that control rats do not engage in risk assessment of cat odors as much. Similar to effects showed by Cooper and Zubek, we show that EE can reinstate behavioral responses that were suppressed due to selective inbreeding. In agreement with our observations, change in environment can unmask behaviors that rats do not normally display in a laboratory cage environment. Thus environment plays a definitive role in mediating behavior.

Previously, we showed that same enrichment paradigm enhances hippocampus dependent contextual processing of fear [8]. The anti-predator response we observe in this study could arise due to stronger contextual processing as well. Similar increase in anti-predator response has been reported earlier in case of familiar environment [66], [67]. Our finding of strong predator aversion becomes more intriguing because enriched animals also showed higher risk-assessment, an active process of searching for predator cue in immediate vicinity rather than mere avoidance of novel environment.

The ventrolateral and dorsomedial part of the ventromedial hypothalamus are known to be involved in reproductive and defensive behaviors, respectively [33]. Our results show for the first time, that neurons of ventromedial hypothalamus undergo changes as a result of exposure to short-term EE in parallel to enhanced adaptive behavior. Hypothlamic neurons are influenced by androgens [34], [68], [69], [70], [71]. The adult brain retains androgen receptors and the sexual dimorphic nature of the VMH, with males exhibiting larger volume [69], [71]. Additionally, VMHvl and hypothalamus-pituitary axis are reciprocally connected, thus diving secretion of androgens and in turn getting influenced by them. In our study, the high testosterone levels of enriched males might drive both reproductive and defensive behavior through neuronal plasticity in the respective hypothalamic region. It is intriguing to note that dorsomedial nucleus undergoes an increase in dendritic length while ventrolateral nucleus undergoes a decrease in the same. This presents a unique puzzle of dendritic plasticity where same independent variable (here EE) causes opposite effects on dendritic architecture in two different hypothalamic regions. However, the phenomenon is not unheard of. Stress similarly induces opposite patterns of dendritic plasticity in the hippocampus (dendritic atrophy) and the amygdala (dendritic expansion) [72].

Our findings reveal an additional interesting correlate. Stress and glucocorticoids can augment dendritic trees in the amygdala [36], increase amygdaloid excitability [73], [74], and enhance amygdaloid-dependent function, including fear conditioning [75], [76], [77]. This suggests that glucocorticoids play a pivotal role in gating the role of the amygdala in emotional responses. In agreement with these observations, we report that EE causes animal to exhibit greater amount of circulating corticosterone in response to cat door, parallel to showing a greater amount of innate fear.

In conclusion, studies concerning EE stretch back a half century [78], [79], and have explored the phenomenon in extensive detail. Despite that, little work has explored the ethological relevance of EE, something addressed in the present paper. We observe that in male rats, EE enhances sexual attractiveness and sexual signaling (i.e., higher testosterone levels), as well as enhancing predator avoidance; moreover, we see logical opposing effects of EE on neuronal cytoarchitecture in two relevant hypothalamic nuclei. The possibility that these two sets of changes are plausibly coupled (i.e., that increased predator avoidance is an adaptive compensation for the conspicuousness of increased sexual attraction) is exciting, and awaits further study.
